# Breech Presentations

**Published:** 1894-07

**Authors:** 


Breech Presentations.—Etienne reports a series of fifty breech labors, with viable fetuses, with no infantile mortality—a remarkable result, considering the usually accepted mortality of 10 per cent., or even 25 to 33 per cent. (Hegar) in primiparous cases. Etienne's cases were conducted in the Nancy lying-in hospital between 1883 to 1891; there were seventy-six cases in all; but twenty-six were rejected in which the fetus was either dead antepartum or non-viable. The secret of the success in the Nancy clinic is a skilfully exerted suprapubic pressure during the extraction, whereby the extension of the head and the slipping up of the arms are prevented. This is no new manœuver; it has long been taught in the best schools, and its importance is occasionally emphasized in journal articles. It is probable that the usual mortality, while partly due to a general want of obstetric skill, is almost entirely attributable to the want of intelligently applied *vis a tergo* while the operator is making traction on the child's legs and trunk. Unquestionably well directed pressure in the proper axis on the fundus uteri through the abdominal walls will almost invariably prevent the extension of the head and the upward displacement of the arms; and consequently it should be an invariable rule of practice that the obstetrician should have with him, during the second stage of breech cases, a skilled assistant. It is not enough to send for assistance after the arrest of the head has taken place, for then it is too late. We are confident that if the above rule is conscientiously followed, the fetal prognosis in breech cases will be greatly improved.—*Columbus Medical Journal.*

